# Inhibiting Amikacin Resistance in Multidrug-Resistant Bacteria with Cadmium and Pyrithione

**DOI:** 10.1007/s00284-025-04372-1

**Published:** 2025-07-16

**Authors:** Angel J. Magaña, David Ngo, Kenneth Burgos, Carolina Dominguez Maldonado, Omniya Abdelmaksoud, Jan Sklenicka, Tung Tran, Fernando Pasteran, Verónica Jimenez, María S. Ramirez, Marcelo E. Tolmasky

**Affiliations:** 1 Center for Applied Biotechnology Studies, Department of Biological Science, College of Natural Sciences and Mathematics, California State University Fullerton, Fullerton, CA 92831 USA; 2 Instituto Nacional de Enfermedades Infecciosas, Antimicrobial Service of the National Institute of Infectious Diseases (ANLIS Dr. Carlos G. Malbran), Buenos Aires, Argentina

## Abstract

The ongoing antibiotic resistance crisis is one of the most pressing public health challenges. Multidrug-resistant bacterial pathogens are reaching the point where some are becoming untreatable. Consequently, besides discovering novel antibiotics, alternative strategies must be explored to manage the problem. One approach is developing inhibitors that overcome resistance to antibiotics currently in use. Resistance to aminoglycosides such as amikacin is mainly due to aminoglycoside-modifying enzymes. Despite being refractory to most resistance enzymes, the semisynthetic amikacin is inactivated by aminoglycoside 6′-*N*-acetyltransferases type I [AAC(6′)-I], of which AAC(6′)-Ib is the most common in Gram-negative pathogens. The discovery that certain divalent and monovalent cations interfere with enzymatic acetylation catalyzed by AAC(6′)-Ib opens possibilities for developing formulations combining antibiotics with these cations to enhance efficacy. Addition of CdCl₂ to in vitro enzymatic assays inhibited transfer of an acetyl group to the 6′-*N* position of amikacin, kanamycin, and tobramycin. Hence, Cd^2^⁺ is a potential adjuvant to aminoglycosides for treating AAC(6′)-Ib-mediated resistant infections. It was initially disappointing that, as with other divalent cations, CdCl₂ addition to cultures of bacteria harboring AAC(6′)-Ib did not reverse resistance. However, the inhibitory action of Cd^2^⁺ became evident when combined with the ionophore pyrithione. The complex efficiently inhibited resistance in *Acinetobacter baumann*ii and *Klebsiella pneumoniae* harboring AAC(6′)-Ib. Furthermore, the combination inhibited amikacin resistance in carbapenem-resistant *K. pneumoniae* clinical isolates. These results add another cation to the arsenal of potential aminoglycoside adjuvants, which could be developed alone or in coordination complexes with ionophores to treat multidrug-resistant infections.

## Introduction

Aminoglycosides are broad-spectrum antibiotics whose basic chemical structures consist of an aminocyclitol nucleus (streptamine, 2-deoxystreptamine, or streptidine) linked to amino sugars through glycosidic bonds [1]. Aminoglycoside antibiotics are used, either alone or in combination with other antimicrobials, to treat a wide range of infections, many of which are life-threatening, caused by both Gram-negative and Gram-positive bacteria. [2, 3]. Although there was a period when the use of these antimicrobials diminished in favor of other classes with lower toxicity, increased multidrug resistance has renewed interest in aminoglycosides [4]. In particular, amikacin, a semisynthetic derivative of kanamycin A obtained by acylation with the l-(−)-γ-amino-α-hydroxybutyryl side chain at the C-1 amino group of the deoxystreptamine moiety of kanamycin A, has been successfully used in multiple treatments due to its refractory nature to most aminoglycoside-modifying enzymes [5]. Unfortunately, amikacin is a substrate of aminoglycoside 6′-*N*-acetyltransferases type I [AAC(6′)-I] enzymes, which is the mechanism of resistance in many bacteria [6]. AAC(6′)-Ib is the most often found in Gram-negative clinical strains [1]. The *aac(6′)-Ib* gene is found in many mobile genetic environments, making it highly mobile and capable of residing in plasmids of different classes and chromosomes [7]. Its ubiquity within Gram-negative pathogens drove efforts to develop compounds that interfere with the acetylating reactions or the enzyme’s expression [2, 8, 9].

Advances in understanding AAC(6′)-Ib-mediated resistance suggest that, at least in cases where the gene is carried on high-copy-number plasmids, the number of enzyme molecules produced far exceeds what is necessary to confer clinical resistance [10]. Therefore, designing or identifying compounds with potent inhibitory activity capable of restoring susceptibility is critical to reverse resistance. Several approaches have been explored to achieve this goal, including the screening of small molecule inhibitors through in-silico molecular docking and combinatorial libraries [11–17], the use of antisense oligomers to reduce resistance by interfering with *aac(6′)-Ib* expression [18, 19], and the discovery that certain cations inhibit the acetylation reaction [20–23].

Divalent cations such as Zn^2+^ and Cu^2+^ efficiently interfere with the enzymatic transfer of the acetyl group to the 6′-*N* position of amikacin and other aminoglycosides. However, when added to culture media at non-toxic concentrations, they do not induce phenotypic conversion to susceptibility in bacteria harboring *aac(6′)-Ib*. Interestingly, when these cations are delivered in complex with ionophores, compounds that facilitate the transport of ions across biological membranes, significantly lower concentrations are sufficient to drastically reduce resistance to susceptibility levels [20–22]. For example, the growth of *Acinetobacter baumannii* or *Escherichia coli* strains carrying *aac*(6′)-*Ib* was significantly inhibited in cultures containing 8 μg/mL amikacin upon the addition of 2 μM Zn^2^⁺ complexed with the ionophore pyrithione [22].

Although the mechanism by which cations interfere with AAC(6′)-Ib-mediated enzymatic inactivation is not yet understood, this property broadens the potential for identifying adjuvant inhibitors that, in combination with amikacin, could successfully treat resistant infections. [20–27]. This article describes the inhibition of AAC(6′)-Ib-mediated amikacin resistance by Cd^2+^ in combination with sodium pyrithione (NaPT).

## Materials and Methods

### Bacterial Strains

*A. baumannii* A155 was isolated from a urinary sample. It is a multidrug-resistant strain belonging to the clonal complex 109 [28]. *Klebsiella pneumoniae* JHCK1 is a multidrug-resistant strain isolated from the cerebrospinal fluid of a neonate [29]. *K. pneumoniae* M27083 is a multidrug-resistant strain that harbors two carbapenemase-coding genes, *bla*_NDM-1_ and *bla*_*KPC-*2_ [30]. It was isolated from mini-bronchoalveolar lavage (mini-BAL) carried out on a male patient with COVID-19. *K. pneumoniae* MA215 is a multidrug-resistant strain that includes *bla*_*KPC-16*2_, isolated from a rectal screening of a female patient [31]. All four strains were isolated in the Autonomous City of Buenos Aires, Argentina, and harbor *aac(6′)-Ib.*

### Acetyltransferase Assays

Acetyltransferase activity was assessed using the phosphocellulose paper binding assay [32], as previously described [33, 34]. Briefly, 120 μg of protein from a soluble extract obtained from sonically disrupted *E. coli* TOP10(pJHCMW1) cells were added to a reaction mixture containing 200 mM Tris HCl pH 7.4 buffer, 167 μM antibiotic, 1 mM cadmium or magnesium chloride, and 0.03 μCi of [acetyl-1-^14^C]-acetyl-coenzyme A (specific activity 60 μCi/μmol) in a final volume of 30 μL. The mixture was incubated at 37 °C for 20 min, and 20 μL were spotted on phosphocellulose paper strips. The unreacted radioactive substrate was washed once by submersion in 80 °C water, followed by three washes with room temperature water. After drying, the phosphocellulose paper strips were placed into scintillation vials containing 4 mL of Ultima Gold scintillation cocktail (Perkin Elmer), and the radioactivity was quantified in a scintillation counter (Beckman LS6500).

### Growth Inhibition Assays

Bacteria were routinely cultured in Lennox L broth (1% tryptone, 0.5% yeast extract, 0.5% NaCl), and 2% agar was added in the case of solid medium. Inhibition of bacterial growth was determined in Mueller–Hinton broth at 37 °C with shaking in microtiter plates. The OD_600_ of the cultures containing the specified additions was determined hourly for 17 h incubation at 37 °C using the BioTek Synergy 2 microplate reader as described before [22]. In the case of the carbapenem-resistant K. pneumoniae (CRKP) assays, the OD_600_ was measured after 20 h. Statistical differences were analyzed using one-way analysis of variance (ANOVA), followed by Dunnet’s multiple comparison test (*P* < 0.05). The analysis was performed using GraphPad Prism (GraphPad Software, San Diego, CA, USA).

### Cytotoxicity Assays

Cytotoxicity was assessed as previously described using HEK293 cells (BEI Resources, Manassas, VA, USA; catalog number NR-9313) [27, 35]. One thousand cells per well were seeded in black microtiter plates and incubated for 12 h before adding the test compound(s). Following compound addition, incubation continued for 24 h. Cells were then washed with sterile D-PBS, resuspended in LIVE/DEAD reagent (2 μM ethidium homodimer-1 and 1 μM calcein-AM; Molecular Probes), and incubated for 30 min at 37 °C. Fluorescence was measured at 645 nm (dead cells) and 530 nm (live cells). The percentage of live cells was calculated relative to untreated controls. Each condition was tested in triplicate, and results were expressed as mean ± SD from three independent experiments. Statistical differences were analyzed using one-way ANOVA with Dunnett’s post-test, comparing each treatment to the control with no compound added.

## Results

### Effect of Cd^2+^ on AAC(6′)-Ib-Mediated Acetylation of Amikacin

The finding that some divalent cations act as inhibitors of AAC(6′)-Ib-catalyzed acetylation of aminoglycosides prompted us to test other metal ions. We found that Cd^2+^ reduced the acetylation of aminoglycosides by AAC(6′)-Ib (Table 1). Mg^2+^, which along with other cations, was shown not to interfere with or enhance enzymatic acetylation [22], was included in the assay as a negative control.

**Table 1:** Effect of CdCl_2_ on acetylation of aminoglycosides catalyzed by AAC(6′)-Ib

	Acetylation (dpm)^a^		
Addition	Aminoglycoside substrate		
	Amikacin	Kanamycin	Tobramycin
None	2572 ± 115	13,010 ± 171	7247 ± 142
CdCl_2_	284 ± 37	1614 ± 83	998 ± 50
MgCl_2_	1940 ± 40	11,733 ± 531	6265 ± 123

^a^Assays were carried out using the phosphocellulose paper method

### Effect of Cd^2+^ and Pyrithione on Amikacin-Resistant Bacteria Harboring ***aac(6′)-Ib***

**Fig. 1:**
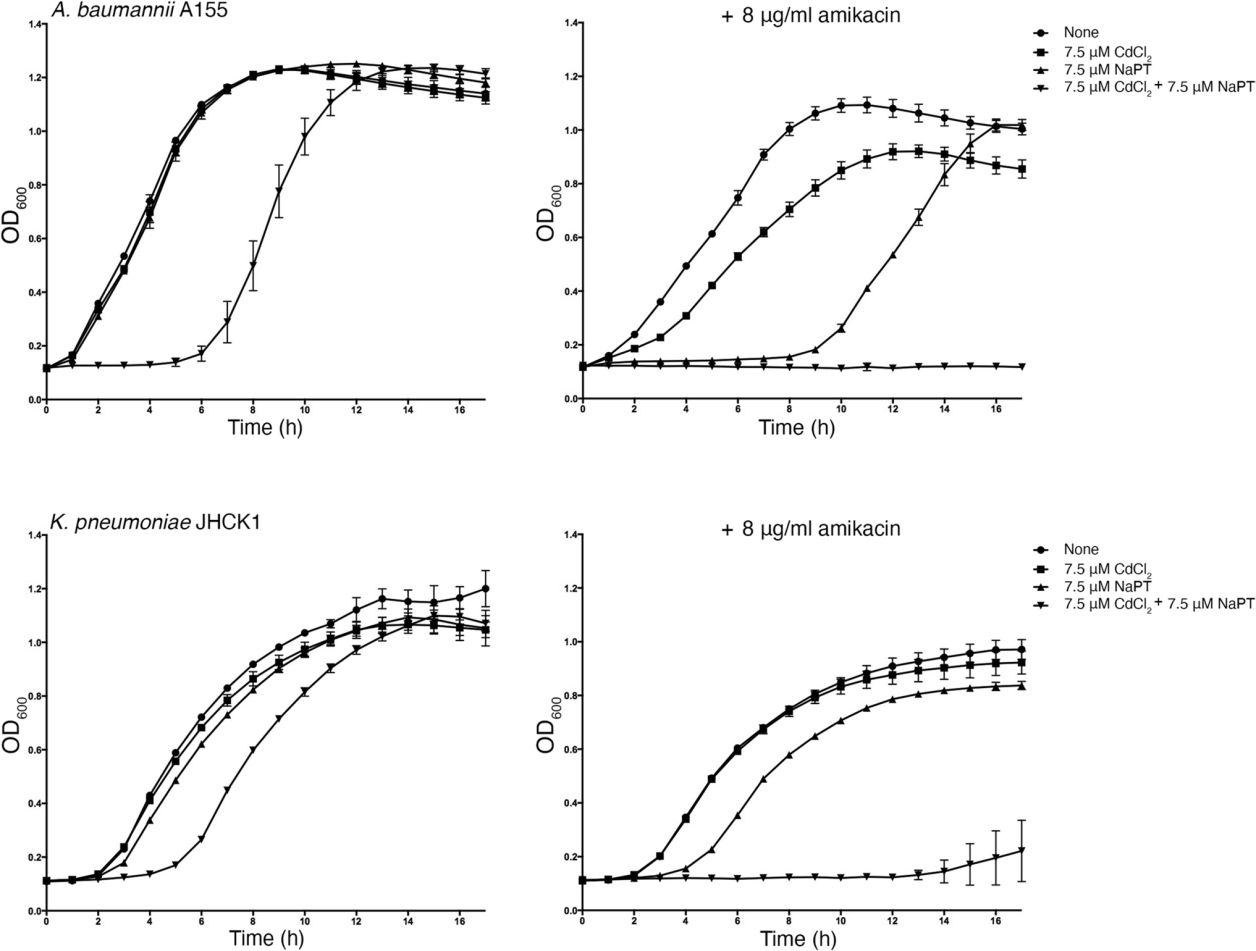
Effect of Cd^2+^ and pyrithione on AAC(6′)-Ib-mediated resistance to amikacin. *A*. *baumannii* A155 and *K. pneumoniae* JHCK1 were cultured in microtiter plates at 37 °C with the additions indicated in the figure. Optical density at 600 nm (OD_600_) was measured hourly. The left panels show growth curves of cultures without amikacin; the right panels show cultures containing 8 μg/mL amikacin. Values are the average from four assays carried out in duplicates

We carried out cultures with different supplements to determine if the effect of Cd^2+^ on the enzymatic reaction catalyzed by AAC(6′)-Ib reduces resistance in bacterial cells growing in broth. The assays were carried out using *K. pneumoniae* JHCK1 and *A. baumannii* A155. Both clinical strains naturally harbor *aac(6′)-Ib*, the latter within the chromosome and the former in a plasmid, pJHCMW1, whose copy number is ~ 25, when measured in *E. coli* [36]. Figure 1 shows that amikacin has minimal effect on the growth of both strains, and addition of CdCl_2_ only marginally enhanced the action of amikacin. On the other hand, when the growth medium was supplemented with amikacin, CdCl_2_, and NaPT, growth was completely abolished. Inspection of the *A. baumannii* A155 growth curves indicates that NaPT influences growth, but it is mainly limited to the extension of the lag phase and not the final cell density after 17 h incubation. The same but significantly reduced effect can be seen in the *K. pneumoniae* JHCK1 growth curves when NaPT is present. Extension of the lag phase in the presence of PT on various bacteria has been documented before [37]. The results described in this section show that 7.5 μM Cd^2+^ in the presence of the ionophore pyrithione reverses the chromosome- and plasmid-mediated resistance to amikacin in *A. baumannii* and *K. pneumoniae* clinical strains.

### Effect of Cd^2+^ and Pyrithione with ***aac(6′)-Ib***-Mediated Resistance to Amikacin in Multidrug-Resistant Bacteria Harboring Carbapenemases

**Fig. 2:**
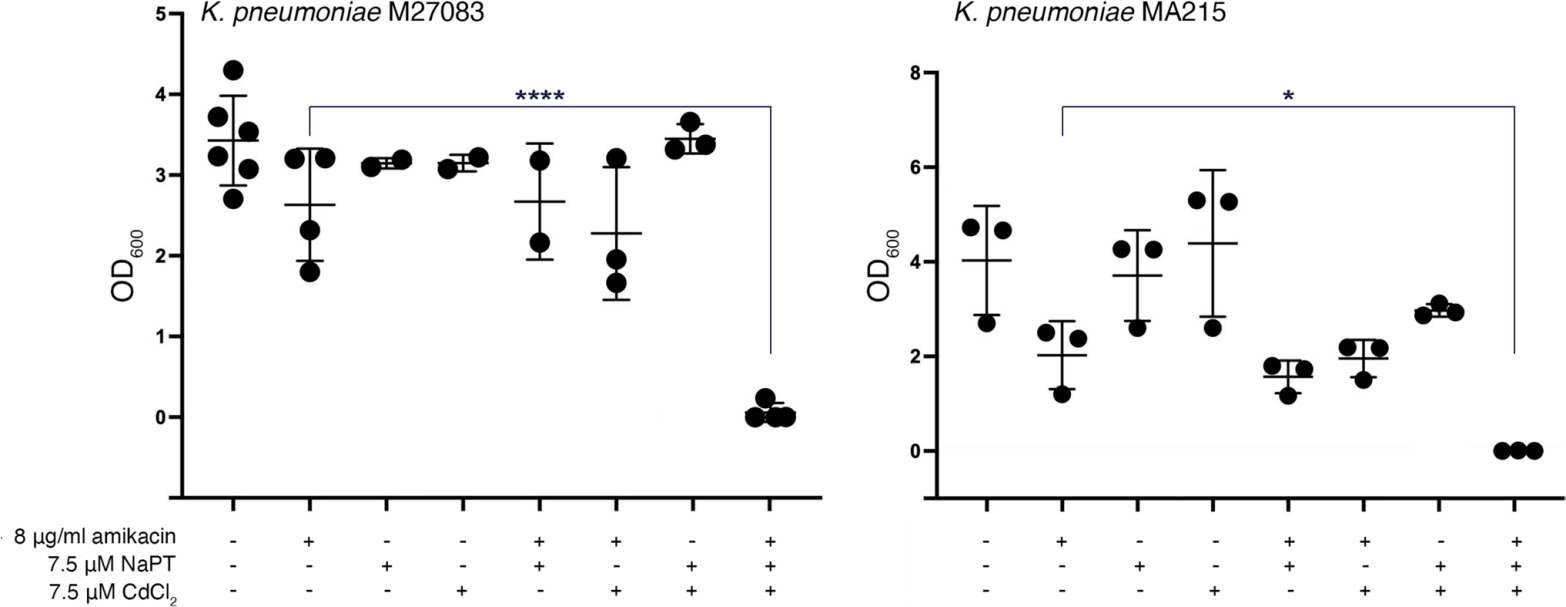
Effect of Cd^2+^ and pyrithione on resistance to amikacin in CRKP strains. *K*. *pneumoniae* M27083 and MA215 were cultured in the presence of the supplements indicated in the figure. After inoculation, the cultures were incubated at 37 °C, and the OD_600_ was determined after 20 h. Growth in the presence of all three supplements was significantly lower than that without additions. Statistical differences were analyzed using one-way analysis of variance (ANOVA), followed by Dunnet’s multiple comparison test (*P* < 0.05)

Among the various types of β-lactam antibiotics, carbapenems have the broadest spectrum and greatest potency against Gram-positive and Gram-negative bacterial pathogens. These characteristics make carbapenems a crucial tool in combating life-threatening, multiresistant infections. Consequently, they are reserved as last-resort treatment options when other therapies have failed [38]. Unfortunately, the rise of several carbapenemases threatens our ability to treat many infections, leading to increased morbidity and mortality [39]. Carbapenemases are rapidly spreading among Enterobacterales, exacerbating the already severe problem of multidrug-resistant nosocomial infections [39]. A significant example of this crisis is the emergence of CRKP strains, which have such a high impact that they have been placed at the top of the World Health Organization’s priority list [40, 41]. We selected two CRKP strains, M27083 and MA215, resistant to amikacin and harboring the *aac(6′)-Ib* gene to determine if the combination of Cd^2+^ and pyrithione induces phenotypic conversion to susceptibility. Figure 2 shows that addition of 8 μg/mL amikacin did not preclude significant growth, although strain MA215 showed slightly lower amikacin resistance levels than strain M27083. In both cases, adding 7.5 μM Cd^2+^ or NaPT to the cultures containing amikacin did not affect growth. However, when both compounds were present, growth was completely abolished. Supplementing the medium with Cd^2+^, NaPT, or both compounds without adding amikacin resulted in heavy bacterial growth.

### Cytotoxicity of the Compounds Tested

**Fig. 3:**
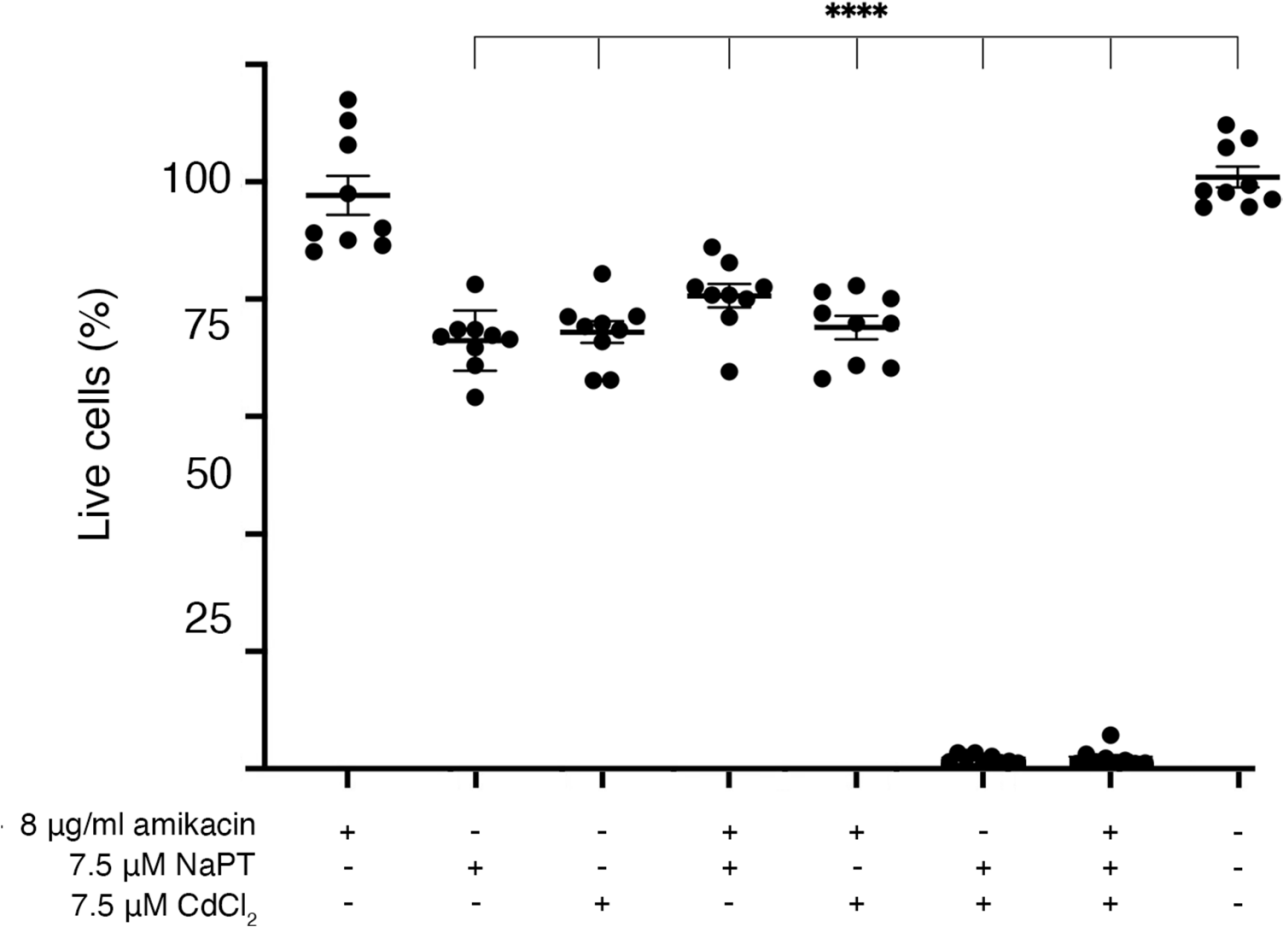
Cytotoxicity of CdCl_2_, NaPT, and amikacin. The cytotoxicity of the compounds and mixes was assessed on HEK293 cells using a LIVE/DEAD kit as described in the Materials and Methods section. The percentage of surviving cells was calculated relative to cells untreated (rightmost test). Assays were carried out in triplicate and the values are mean ± SD of three independent experiments. Statistics were determined using one-way ANOVA with Dunnett’s post-test comparing each treatment to the control, four asterisks indicate *P* < 0.0001

Low toxicity is a desirable property of therapeutic tools for systemic use. However, medicines can still be useful in localized treatments despite being too toxic for systemic use. We carried out an analysis of the cytotoxicity of the compounds tested in this work using HEK293 cells. Figure 3 shows that amikacin did not show toxicity at the concentrations used in this work. On the other hand, both compounds, CdCl_2_ and NaPT, showed low but significant toxicity, and the combination of CdCl_2_ and NaPT was highly toxic.

## Discussion

The ongoing antibiotic resistance crisis ranks among modern medicine’s most significant challenges [40, 42]. Unfortunately, the optimistic view that we were on the verge of defeating bacterial infections, prevalent decades ago, never crystallized. The current rise and dissemination of multidrug-resistant pathogens threaten to undermine much of the progress made in treating infectious diseases. In the past, a robust pipeline of new antibiotics ensured that when resistance to existing drugs became critical, there was always a new one to control infections. However, a combination of scientific challenges and economic considerations has slowed the pace of discovery of new antimicrobials, allowing rapidly evolving bacteria to outpace our efforts [43]. Some bacteria now resist most or all available antibiotics, creating a dangerous gap in medicine’s ability to combat life-threatening infections. It is, therefore, imperative to complement antibiotic discovery with innovative approaches such as alternative therapeutic strategies, repurposing of existing compounds, or agents that can overcome resistance to antibiotics currently in use [40].

Aminoglycoside antibiotics, alone or in combination with other antimicrobials, are effective in treating numerous infections caused by Gram-negative aerobic bacilli, staphylococci, and other Gram-positives [1]. The most common mechanism of resistance to aminoglycosides is their enzymatic modification [1]. To date, more than one hundred aminoglycoside-modifying enzymes have been described. Among them, AAC(6′)-Ib stands out for its ubiquity among Gram-negative pathogens and its effectiveness in conferring resistance to numerous aminoglycosides. AAC(6′)-Ib is the most prevalent enzyme that causes resistance to the semisynthetic aminoglycoside amikacin [7]. This antibiotic has been instrumental in controlling numerous otherwise multidrug-resistant infections. Finding strategies to overcome resistance mediated by AAC(6′)-Ib would extend the effective lifespan of amikacin and permit its continued use. A promising and recent finding in the quest for alternatives to overcome the action of AAC(6′)-Ib is the ability of some cations to interfere with the acetylation reaction. Cations with inhibitory properties include Zn^2+^, Cu^2+^, and Ag^1+^; the divalent ones are active in growing bacterial cells when combined with an ionophore such as pyrithione [20–23]. The quest for improvement and refinement of methods to overcome amikacin resistance led to the identification of Cd^2+^ as another cation that interferes with enzymatic acetylation-mediated inactivation. The results presented in this article demonstrate that, in addition to its in vitro activity, low micromolar concentrations of Cd^2+^ in the presence of pyrithione completely inhibited the growth of AAC(6′)-Ib-harboring *A. baumannii* and *K. pneumoniae* clinical isolates. We hypothesize that Cd^2+^ and PT may form a complex that facilitates cation uptake, leading to inhibition of acetylation at levels sufficient for conversion to susceptibility at the tested amikacin concentration. Alternatively, pyrithione may enhance Cd^2^⁺ internalization without forming a complex. Interestingly, the growth of two CRKP strains that harbor AAC(6′)-Ib was effectively controlled by amikacin in the presence of pyrithione and Cd^2+^. This work expands the repertoire of potential inhibitors of the enzymatic inactivation of amikacin. These compounds could be developed, alone or combined with other inhibitors, as adjuvants to be administered with amikacin to treat the most severe multidrug-resistant infections.

While Cd^2^⁺ exhibits significant toxicity to biological systems and the combination with pyrithione was highly toxic, its ability to enhance amikacin’s efficacy in combination with sodium pyrithione could be used in topical formulations. Topical administration minimizes systemic absorption, thereby reducing toxicity risks while exerting localized therapeutic action [44]. Amikacin in topical ointment formulation is already commercially available, and the use of the antibiotic in topical form has been reported for various treatments like *K. pneumoniae* keratitis or *Mycobacterium marinum* skin infections [45, 46]. The viability of localized amikacin delivery supports the feasibility of developing more robust formulations that include Cd^2^⁺-based potentiators.

While the mechanism by which cations inhibit acetylation remains to be determined, an attractive hypothesis is that the cation titrates the substrate aminoglycoside by forming coordination complexes. This process has been described to occur between aminoglycosides and several metal ions [47].

Future experiments will focus on elucidating the mechanism of cation-mediated inhibition and identifying combinations of inhibitors, including other cations, small molecules, or antisense oligomers targeting *aac(6′)-Ib* expression, that can reverse resistance in a broad range of amikacin-resistant Gram-negative pathogens.

## Conclusions

Only inhibitors of β-lactamases have reached the market to be used in combination with β-lactams [48]. The success in extending these antibiotics’ usability demonstrates the strategy’s viability. Unfortunately, no resistance inhibitors for other antibiotic classes have been successfully developed. On the other hand, the recent discovery that various cations inhibit the resistance mediated by AAC(6′)-Ib is encouraging and offers a potential path for the continued use of amikacin and other aminoglycosides for hard-to-treat multidrug-resistant infections. The results described in this article expand the armamentarium of cations that can be developed into adjuvants to amikacin and other aminoglycosides.

## Data Availability

All data generated or analyzed during this study are included in this published article.
